# Favorable response to surufatinib in a patient with necrolytic migratory erythema: A case report

**DOI:** 10.1515/biol-2022-0672

**Published:** 2023-11-27

**Authors:** Zhongan Liu, Feng Hu, Shuang Guo, Peng Zhang, Guiling Li, You Qin

**Affiliations:** Cancer Center, Union Hospital, Tongji Medical College, Huazhong University of Science and Technology, Wuhan 430022, China; Department of Dermatology, Wuhan No. 1 Hospital, Wuhan 430022, China; Department of Pathology, Union Hospital, Tongji Medical College, Huazhong University of Science and Technology, Wuhan 430022, China

**Keywords:** necrolytic migratory erythema, pancreatic neuroendocrine tumors, surufatinib

## Abstract

Necrolytic migratory erythema (NME) is usually associated with paraneoplastic syndrome caused by functional pancreatic neuroendocrine tumor (PNET). Accurate diagnosis and effective treatment of NET-related NME is challenging due to its rarity and lack of typical clinical symptoms and specific pathological manifestations. Here we report a rare case of PNET with NME as the initial manifestation. 68Ga-DOTA-TATE PET/MR was used to detect the primary pancreatic and metastatic liver tumors. Finally, the patient was diagnosed as PNET via liver biopsy. After four cycles of standard capecitabine plus temozolomide chemotherapy combined with long-acting octreotide, the patient’s skin lesions on both lower extremities improved only slightly, while tumors remained stable and unchanged in size. Then the patient was treated with surufatinib. Two months later, the skin lesions healed completely, and tumors responded significantly. This rare case suggests that surufatinib may be a promising therapy for patients with PNET-associated NME.

## 1 Introduction

Pancreatic neuroendocrine tumor (PNET) is a highly heterogeneous malignant disease, with diverse biological behaviors depending on the specific pathological type, clinical stage, differential grade, tumor burden, and histological characteristics [1]. PNET can be broadly divided into functional and non-functional PNET subtypes according to their clinical features and endocrine hormones. Functional tumors exhibit complex clinical symptoms based on tumor cell-produced hormones, including insulin, gastrin, glucagon, and somatostatin [2].

Necrolytic migratory erythema (NME) is currently considered to be a rare cutaneous paraneoplastic syndrome caused by pancreatic islet alpha cell tumors, which is mainly characterized by recurrent migratory necrolysis ring or annular dark erythema [3]. NME usually begins as an itchy and painful erythema that gradually enlarges and merges to form bullous and ulcerative lesions surrounded by brown pigmentation [4]. Its exact pathogenesis remains unclear. At present, most views believe that it is related to hyperglucagonemia, amino acid deficiency, zinc deficiency, etc. [5]. NME may be a specific or initially identified symptom of PNETs. Clinically, PNETs are often overlooked due to the rarity of NME and the limitations of imaging studies.

Currently, there is no standard treatment for PNET-related NME. Pancreatic tumor resection has always been the most effective treatment for local or regional lymph node disease. However, due to rapid tumor progression, most patients are diagnosed at an advanced stage. Among them, 60% of patients have liver metastases at diagnosis [6]. For recurrent or unresectable disease, hepatic interventional arterial embolization or radionuclide therapy can relieve the symptoms of hyperhormonal secretion in patients by reducing the volume of liver metastases. The role of somatostatin analogs in controlling clinical symptoms of functional neuroendocrine tumors has been confirmed, but it cannot achieve the purpose of inhibiting tumor progression [7,8]. The emergence of small-molecule targeted drugs such as everolimus and sunitinib has provided new treatment options for these patients [9,10]. However, due to the emergence of drug resistance and the limitation of clinical application, the therapeutic effect of this type of tumor is not ideal. Therefore, it is particularly important to find a new type of treatment with low toxicity, high efficiency, and high remission rate.

Surufatinib is a novel small molecule inhibitor that simultaneously inhibits tumor angiogenesis and suppresses immune evasion through colony-stimulating factor-1 and fibroblast growth factor signaling pathways, completely breaking small molecules such as sunitinib and everolimus limitations of clinical application of targeted drugs [11]. Two clinical studies, SANET-ep and SANET-p, confirmed that surufatinib can significantly improve the progression-free survival of patients in both advanced PNETs and extra PNETs, and has a good risk–benefit ratio. Unfortunately, neither study included patients with PNETs with NME [12,13].

Here we report a rare case of PNET with NME as the first symptom that was finally confirmed by PET/MR and pathology. Tumors responded excellently to second-line surufatinib treatment, with complete healing of both lower extremity lesions, suggesting that surufatinib therapy could be a promising treatment option for PNET complicated with NME.

## 2 Case presentation

**Figure 1:**
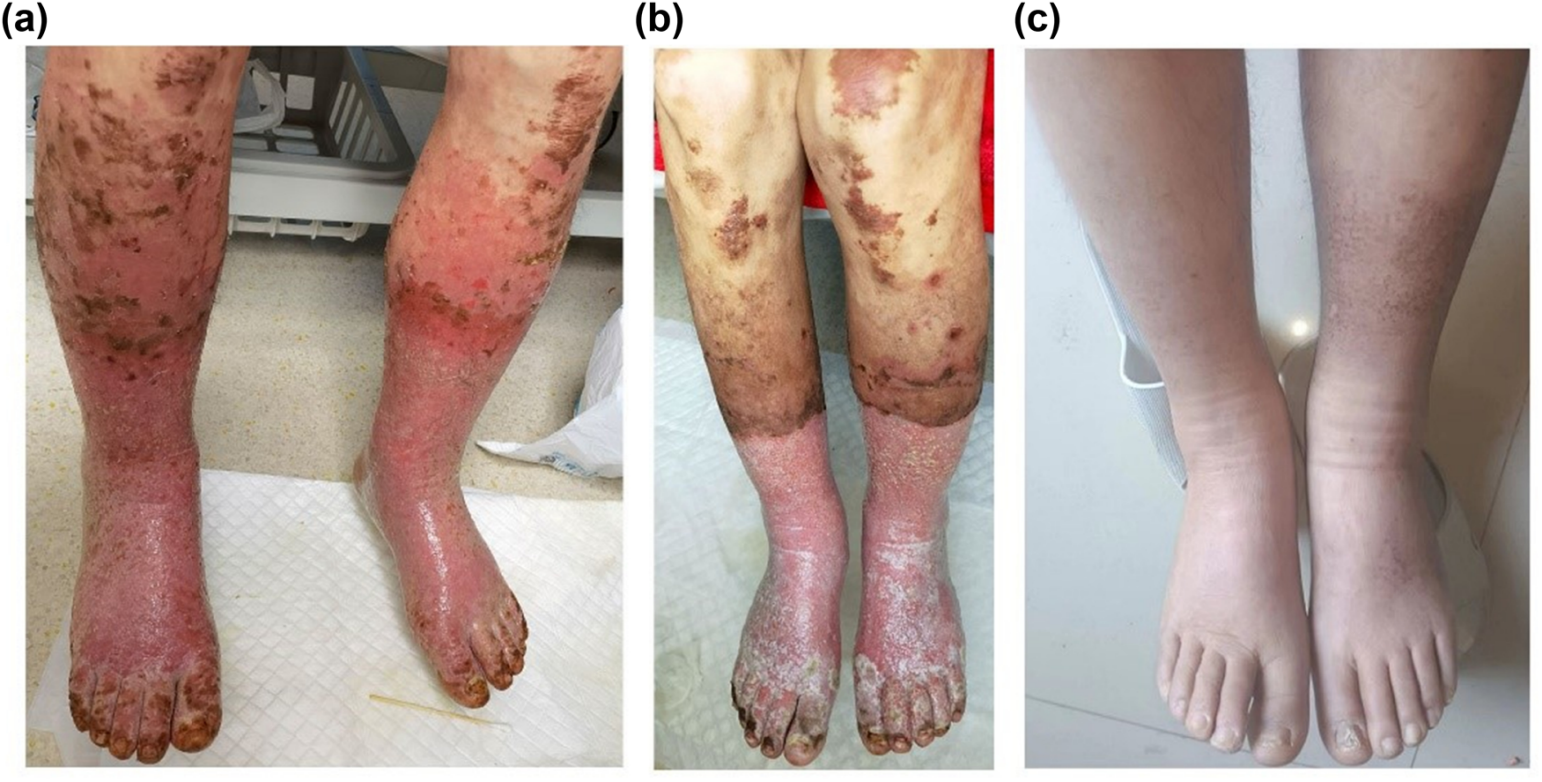
Skin appearance of the patient. (a) Both calves and dorsum of both feet were edemata, large infiltrative erythema, covered with white scales, flaky erosions on both feet, and large areas of maceration on both feet. (b) After four cycles of oral temozolomide combined with capecitabine, the lower extremity skin edema and pain symptoms improved slightly. (c) After four cycles of surufatinib treatment, the lower extremity ulcers and pain symptoms disappeared, and normal skin tissue grew.

A 33-year-old male patient presented to our hospital with complaints of recurrent peripheral rash for nearly 3 years, and bilateral lower extremity edema for 1 month. Initially, the patient started to develop a skin rash on both lower extremities in July 2018 without any specific apparent reasons. He tried a variety of treatments, but with little improvement in clinical symptoms. Conversely, the disease progressed slowly. In early June 2021, the patient developed bilateral lower limb edema with molting-like changes. A clinical examination showed multiple scattered rashes and plaques all over the body, partially fused into patches, with surface pigmentation and occasional pruritus. The epidermis of both lower extremities was sock cover-like necrotic and exfoliated, with a large amount of yellowish fluid exuding. Meanwhile, he complained of pain in both lower extremities, seriously affecting his quality of life (Figure 1a).

**Figure 2:**
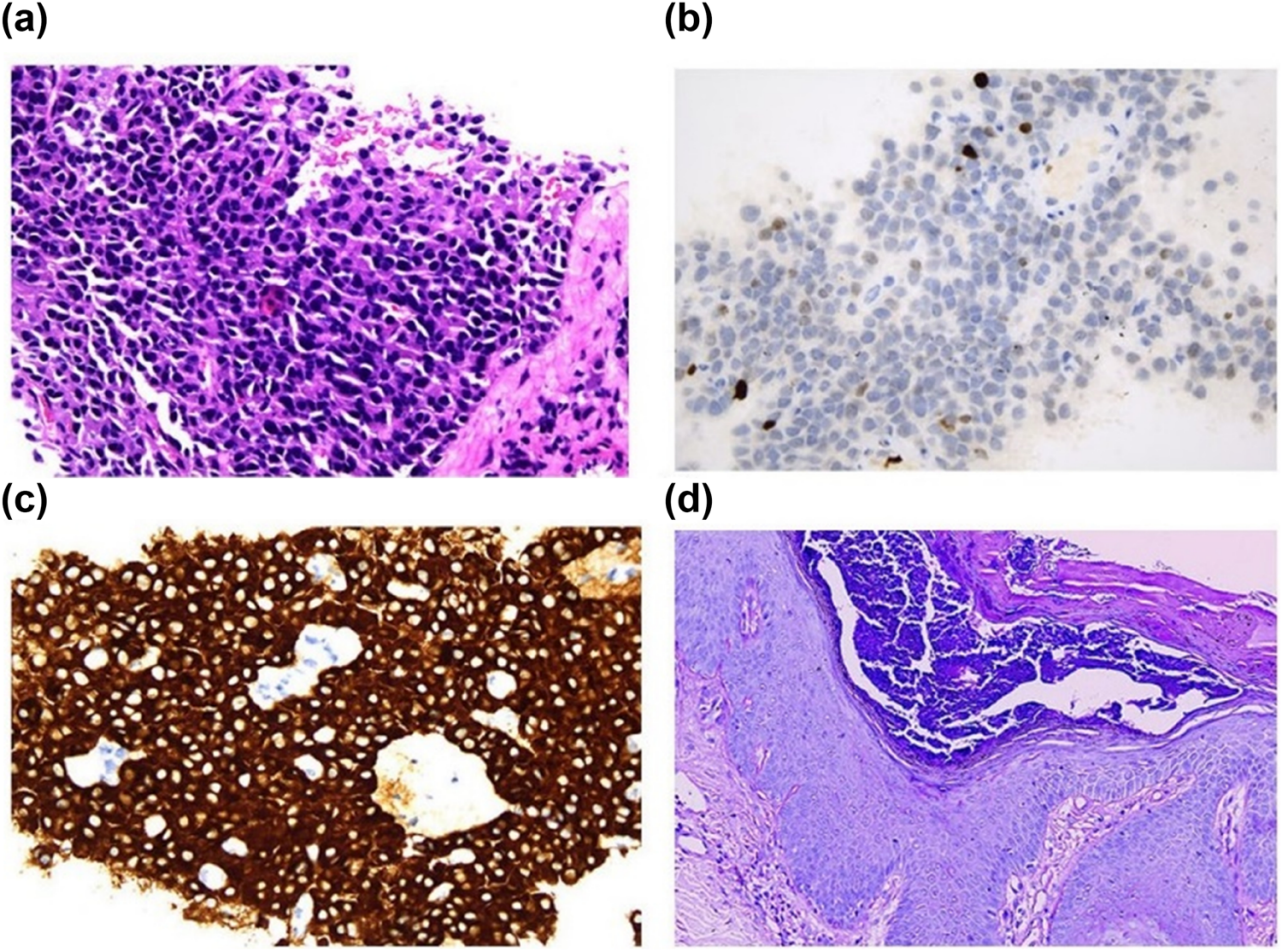
Patient’s liver biopsy and skin histological findings. (a) Liver biopsy results suggest neuroendocrine tumor. (b) Results of ki-67 staining indicated that the value-added index was about 3%. (c) Immunohistochemistry showed positive for Syn protein. (d) Skin pathology of the patient showed hyperkeratosis, fusion keratosis imperfect, subcortical pustules, “psoriasis-like” hyperplasia of the epidermis, and different amounts of lymphocyte infiltration in the superficial dermis.

**Figure 3:**
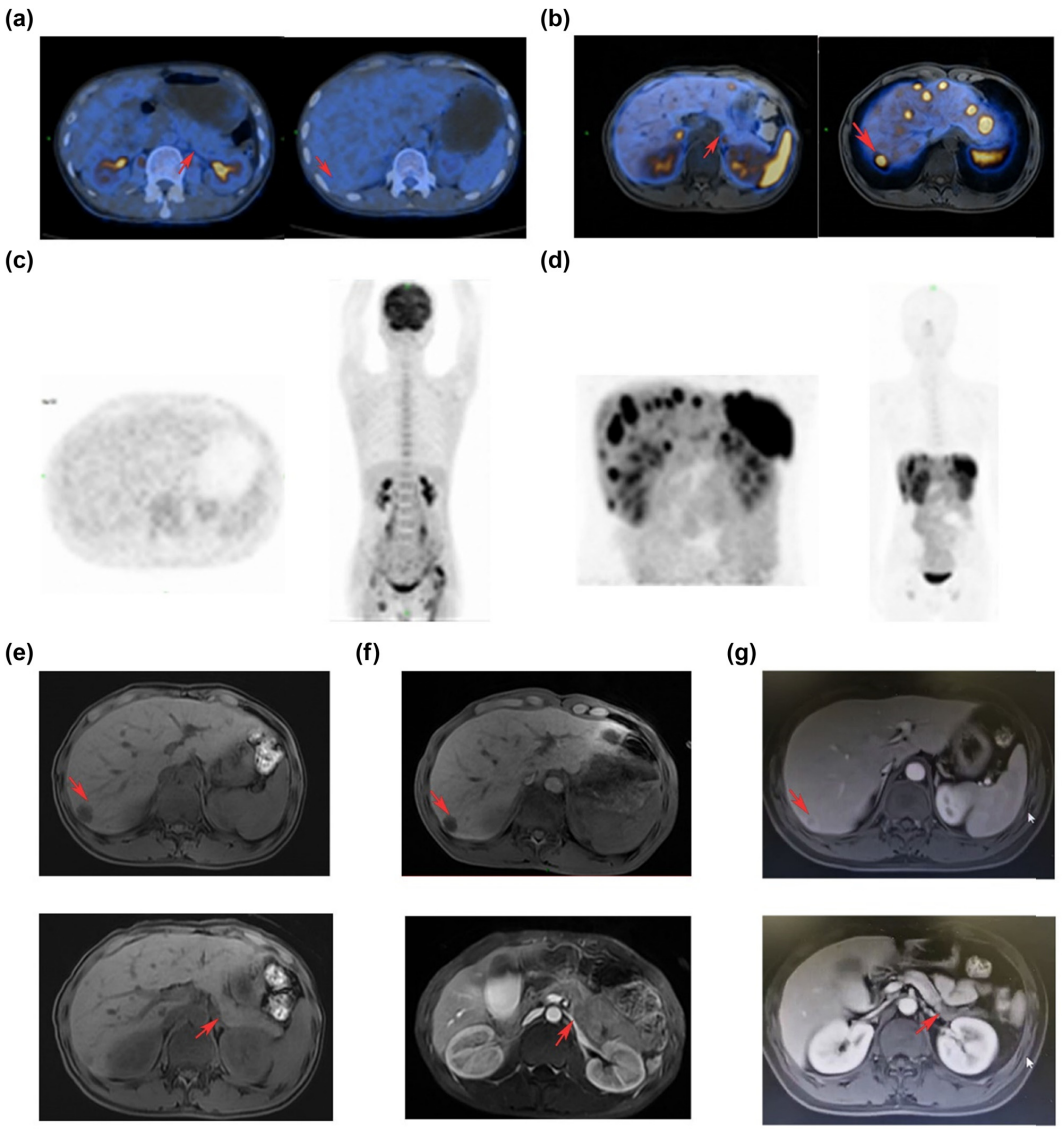
Manifestation of tumor on 68Ga-DOTA-TATE/PET and 18F-FDG PET/CT imaging and the images of patients after treatment. (a, c) PET-CT images of the patient, no obvious hypermetabolic lesions were found. (b, d) PET/MR images of the patient, the distribution of imaging agents in the body and tail of the patient’s pancreas and liver is abnormally concentrated. (e) Liver and pancreas lesions at the patient’s initial diagnosis. (f) MR image of the patient after four cycles of chemotherapy, indicating that the liver and pancreas lesions did not change significantly compared with the previous ones. (g) Patient was re-examined after four cycles of oral surufatinib, suggesting that the liver and pancreas lesions continued to shrink.

Considering the patient’s unexplainable skin symptoms and weight loss in the past 6 months, we performed an abdominal MR on the patient and found a mass of about 14 mm in the head of the pancreas and multiple liver masses, which may be pancreatic tumors combined with liver metastasis. To further clarify the staging and diagnosis, we arranged PET/CT examination for him, it reported that there are multiple low-density shadows in the liver, considering the possibility of malignant tumor metastasis, and multiple lymph nodes in the abdominal cavity, the largest diameter of which is about 2.6 × 1.7 cm, but their standardized uptake value (SUV) is not high, we consider the possibility of lymphadenitis. Interestingly, the body of the pancreas was slightly distended locally, but no hypermetabolic lesions were found in the pancreas (Figure 3a and c). In view of the patient’s special metabolic performance of 18F-FDG, imaging experts suggest that somatostatin receptor imaging is more helpful to clarify the origin and nature of the tumor in patients. Therefore, the patient’s 68Ga-DOTA-TATE/PET followed. We could see that the distribution of imaging agents in the body and tail of the patient’s pancreas and liver is abnormally concentrated, which is considered to be a somatostatin receptor-positive tumor, and the skin and subcutaneous tissue of bilateral calves are considered to be highly likely to be inflammatory (Figure 3b and d). The patient’s fasting blood glucose and insulin were all within the normal range, tumor markers such as CEA, PSA, SCC, and NSE were slightly elevated. Combined with the patient’s clinical and imaging examinations, we could obtain a preliminary clinical diagnosis that PNET is associated with multiple liver metastases, and the skin lesions are likely to be NME. The final pathological result of the patient is particularly important for our initial judgment, considering the specificity of the anatomical location of the pancreas and the risks associated with a puncture, we performed an ultrasound-guided biopsy of liver metastases on August 18, 2021 (Figure 2a) (Table 1). The diagnosis of neuroendocrine tumors (G2, NCCN 2019) was supported according to the HE and immunohistochemistry. The histological findings of the skin further support our diagnosis (Figure 2b).

**Table 1:** Final results of IHC

IHC	Result
PCK	(+)
CK8/18	(+)
CgA	(+)
Syn	(+)
CD56	(+)
β-Catenin	(Membrane+)
PR	(+)
E-Cadherin	(+)

Ki-67 protein is a cellular marker for proliferation; CD, cluster of differentiation; PCK, pan cytokeratin; E-Cadherin, epithelial cadherin; CK, markers of visceral glandular epithelial tumors; CgA, chromogranin A; Syn, synapsin – a fibrillar phosphoprotein present in axon terminals, often positive in neuroendocrine tumors.

Although surgical treatment is mainly used for PNET with NME, patients have multiple metastases of liver and abdominal lymph nodes, so more attention should be paid to fluid balance, lower extremity skin care, and primary tumor control [14,15]. For lower extremity edema, we treat it with sodium and potassium supplementation, albumin supplementation, and maintenance of water–electrolyte balance, and for lower extremity skin symptoms, pain relief, lidocaine wet compresses, topical epidermal growth factor, and anti-infection treatment. The skin symptoms of the patient were considered to be related to the tumor, so octreotide was subcutaneously injected, and continued oral capecitabine in combination with temozolomide outside the hospital. The chemotherapy regimen consisted of oral capecitabine, 1,000 mg/m^2^ twice daily for 14 days (days 1–14), and oral temozolomide, 200 mg/m^2^ once daily for 5 days (days 10–14), every 28 days. The patient had received seven cycles of combined chemotherapy until December 2021. Considering that the symptoms of the patient’s lower extremities and tumor regression were not significantly improved, we changed the treatment plan to oral surufatinib in December 2021. At present, the patient’s quality of life has been significantly improved, and the tumor is still regressing (Figures 1b and c and 3e–g).


**Informed consent:** Informed consent has been obtained from all individuals included in this study.
**Ethical approval:** The research related to human use has been complied with all the relevant national regulations, institutional policies and in accordance with the tenets of the Helsinki Declaration, and has been approved by the authors’ institutional review board or equivalent committee.

## 3 Discussion

Functional PNETs account for approximately 30–40% of all PNETs. Unlike nonfunctional tumors, the underlying pathogenesis of the usual clinical presentation relies on the tumor’s abnormal production of protein hormones, proteins, and other substances. NME is a rare paraneoplastic syndrome of functional PNETs, generally thought to be associated with glucagonomas from islet alpha cells [16]. Clinically, the shin lesions are characterized by migratory scaly erythematous papules, patches, and plaques exhibiting super epithelial necrosis, central flaccid bullae, and crusting [3,4,17]. Lesions similar to NME have also been reported in some diseases such as liver diseases, malnutrition, chronic pancreatitis, and inflammatory bowel disease [18,19]. NME is often the first clinical finding of an occult neuroendocrine pancreatic neoplasia. Because of the atypical clinical symptoms and nonspecific pathological manifestations, it brings great difficulties to the disease diagnosis of patients. Clinically, it is often misdiagnosed as drug eruption, urticaria vasculitis, aphthous ulcer, and seborrheic dermatitis [20,21]. In this case, the patient began to develop a skin rash on both lower limbs in July 2018. The local hospital diagnosed it as skin eczema or drug rash. Since the local symptoms of the patient were mild and the skin symptoms of the patient improved slightly after symptomatic treatment, the patient did not receive further skin tissue biopsy and relevant imaging examination. After admission, the patient was finally diagnosed as a PNET with necrotizing migratory erythema after two liver biopsies and pathological consultation in several hospitals. Unfortunately, we did not obtain pathological results for the location of the primary pancreatic lesion.

As a special type of tumor, most neuroendocrine tumors express somatostatin receptors, and that can be used as a target for radionuclide imaging [22]. Based on the differential expression of growth inhibitor receptors in tumor tissues, 68Ga-DOTA-TATE/PET is more likely to reveal functional tumor volume, which is essential for early prediction of treatment outcome and adjustment of treatment regimens. Different from metabolism-based tracers such as 18F-FDG, 68Ga-DOTA-TATE/PET can more accurately reflect the tumor biological behavior of such patients, and it also has higher sensitivity, specificity, and lower radiance rate. Panagiotidis et al. [23] evaluated the impact of 68Ga-DOTA-TATE/PET and 18F-FDG PET/CT-based clinical treatment decisions in patients with neuroendocrine tumors, showing that SUVmax of 68Ga-DOTA-TATE/PET correlates with tumor grade and Ki67 index and can be used to predict prognosis, while 18F-FDG PET/CT is more appropriate for more aggressive and less differentiated NETs [23]. Clinically, they are often combined to customize a personalized diagnosis and treatment plan for patients. In this report, the patient did not show hypermetabolic lesions on PET/CT, which demonstrates its specific biological behavior. The advantages of functional imaging also provide more basis for patients to choose targeted therapy on the other hand.

The treatment of advanced PNETs is limited. The treatment mainly includes tumor resection, chemotherapy, somatostatin analogs, and radiotherapy. Although the early diagnosis of the disease is very important to the prognosis of patients, 50–90% of patients may have liver metastasis at the time of diagnosis [17,24]. This indirectly makes it almost impossible to perform radical surgery on such patients. However, there is also evidence that patients with liver metastases can be treated with resection for grade 1 or 2 tumors, if at least 30% of the liver will remain and if there is no evidence of non-resectable extrahepatic metastases [15,25]. Somatostatin is an important supplement to systemic therapy for patients. It is a polypeptide produced by paracrine cells of the gastrointestinal tract, which can inhibit the release of hormones such as glucagon, gastrin, and insulin. Adverse reactions may include rapid allergic reactions and anti-elastic hypersecretion after the cessation of treatment. The PROMID study [7] demonstrated that octreotide long-acting release prolongs tumor progression to 14.3 months versus 6 months with placebo in functionally active and inactive metastatic midgut NETs.

At present, capecitabine combined with temozolomide has been proved to be effective and well-tolerated in gastrointestinal neuroendocrine tumors [26]. An Eastern Cooperative Oncology Group-sponsored, prospective, randomized, phase 2 trial investigated temozolomide alone versus temozolomide plus capecitabine in 144 patients with progressive G1/G2 pNETs. The combination of temozolomide and capecitabine was associated with a significantly improved progression free survival (PFS) (median PFS, 14.4 months in the temozolomide arm vs 22.7 months in the temozolomide/capecitabine arm) and overall survival (OS) (median OS, 38 months in the temozolomide arm vs not reached in the temozolomide/capecitabine arm) [27]. Although the CAPTEM study has achieved sound clinical treatment effects, more evidence-based medicine is needed to determine whether chemotherapy should be used as a first-line treatment for patients with advanced neuroendocrine tumors.

Targeted therapeutic drugs have made continuous breakthroughs in neuroendocrine tumors recently, including sunitinib, a tyrosine kinase inhibitor and everolimus, an mTOR inhibitor [28]. Sunitinib has achieved good efficacy in pancreatic cancer patients, but with the emergence of multi-target resistance in tumors, its clinical application has more limitations. Surufatinib is a novel small-molecule inhibitor that can simultaneously inhibit tumor angiogenesis and reduce immune escape to target tumors, which fully breaks the limitation of sunitinib resistance. SANET-p was a multicenter, randomized, double-blind, placebo-controlled, phase 3 study, done in 21 hospitals across China [13]. This study aimed to assess the efficacy and safety of surufatinib in patients with advanced pancreatic NETs. The inclusion criteria were mainly for patients with PNETs who progressed after treatment with more than two regimens. The median investigator-assessed progression-free survival was 10.9 months for surufatinib versus 3.7 months for placebo. The study is a great breakthrough for patients with neuroendocrine tumors with limited targeted drugs. Unfortunately, the study excluded all functional neuroendocrine tumors requiring long-acting somatostatin analogs (SSAs). In this case report, the patient was not treated effectively after pre-treatment with oral temozolomide in combination with capecitabine, and despite the use of the same growth inhibitors, both skin symptoms and tumor lesion regression were not significant and were accompanied by more adverse effects. It also suggests that surufatinib in combination with long-acting SSAs can be equally effective in a specific population of functional neuroendocrine tumors and has a broader future in patients with PNETs. Due to the better efficacy and safety of surufatinib, more clinical trials are being carried out, and more patients will benefit from this regimen in the future.

In summary, our report further deepens clinicians’ understanding of PNETs with NME. On the other hand, the status of chemotherapy and targeted therapy in the first-line treatment of advanced neuroendocrine tumors requires more clinical data to support, and the emergence of surufatinib will provide more clinical treatment options for these patients.
